# Big brown bats experience slower epigenetic ageing during hibernation

**DOI:** 10.1098/rspb.2022.0635

**Published:** 2022-08-10

**Authors:** Isabel R. Sullivan, Danielle M. Adams, Lucas J. S. Greville, Paul A. Faure, Gerald S. Wilkinson

**Affiliations:** ^1^ Department of Biology, University of Maryland, College Park, MD 20742, USA; ^2^ Department of Biological Sciences, Towson University, Towson, MD 21252, USA; ^3^ Department of Psychology, Neuroscience and Behaviour, McMaster University, Hamilton, ON, Canada L8S 4K1; ^4^ Department of Biology, University of Waterloo, Waterloo, ON, Canada N3 L 3G1

**Keywords:** DNA methylation, epigenetic clock, *Eptesicus fuscus*, metabolism, immunity, longevity

## Abstract

Comparative analyses of bats indicate that hibernation is associated with increased longevity among species. However, it is not yet known if hibernation affects biological ageing of individuals. Here, we use DNA methylation (DNAm) as an epigenetic biomarker of ageing to determine the effect of hibernation on the big brown bat, *Eptesicus fuscus*. First, we compare epigenetic age, as predicted by a multi-species epigenetic clock, between hibernating and non-hibernating animals and find that hibernation is associated with epigenetic age. Second, we identify genomic sites that exhibit hibernation-associated change in DNAm, independent of age, by comparing samples taken from the same individual in hibernating and active seasons. This paired comparison identified over 3000 differentially methylated positions (DMPs) in the genome. Genome-wide association comparisons to tissue-specific functional elements reveals that DMPs with elevated DNAm during winter occur at sites enriched for quiescent chromatin states, whereas DMPs with reduced DNAm during winter occur at sites enriched for transcription enhancers. Furthermore, genes nearest DMPs are involved in regulation of metabolic processes and innate immunity. Finally, significant overlap exists between genes nearest hibernation DMPs and genes nearest previously identified longevity DMPs. Taken together, these results are consistent with hibernation influencing ageing and longevity in bats.

## 1. Introduction

A variety of mammals save energy during periods of food shortage by entering into torpor, a physiological state characterized by reduced body temperature and metabolic rate that lasts a few hours or days [1,2]. More prolonged bouts of torpor that occur during the winter are referred to as hibernation, and occur in some rodents, bats, carnivores, hedgehogs, marsupials, tenrecs and primates [3–5]. Comparisons of gene expression in tissues taken from hibernating and non-hibernating animals have revealed that hibernation not only suppresses metabolic function [6–9] but also protects skeletal muscle from atrophy [10,11] and inhibits immune function [10,11]. Despite the widespread occurrence of hibernation among mammals, the genetic mechanisms regulating this process remain poorly understood in most species [12,13].

Recently, DNA methylation (DNAm) has been used to gain insight into patterns of gene regulation during hibernation [14,15]. DNA is methylated when a methyl group is added to a cytosine at cytosine–guanine dinucleotide (CpG) sites [16]. While this change can be functionally silent, DNAm can suppress transcription by recruiting proteins involved in gene repression [15,17] or by altering chromatin states to inhibit transcription factor binding [18]. Conversely, loss of DNAm, especially in promoter regions, can permit recruitment of proteins involved in gene activation and result in transcription. Examining patterns of DNAm in a tissue can, therefore, reveal repressed or elevated transcription of specific genes or gene pathways in response to environmental changes [16,19].

To date, DNAm has only been examined in a few hibernating rodent species. Compared to euthermic controls, global DNAm increased nearly twofold in brown adipose tissue [15] but decreased in liver and skeletal muscle in 13-lined ground squirrels, *Ictidomys tridecemlineatus* [14]. The presence of DNAm has also been used to assess tissue-specific transcription in a hibernation candidate gene (*HP-27*) in chipmunks (*Tamias asiaticus*); DNAm at an upstream stimulatory factor (USF)-binding site, which partially regulates transcription of *HP-27*, was reduced in the liver but increased in the kidneys and heart [20], which is consistent with liver-specific transcription for *HP-27*. In yellow-bellied marmots (*Marmota flaviventer*), epigenetic ageing was estimated recently to occur at a faster rate in the active season compared to the hibernation season [21].

Hibernation occurs in many species of bats found in temperate regions [3,4,22] and is of particular interest because it is associated with the evolution of extreme longevity [23]. Here, we used big brown bats, *Eptesicus fuscus*, to investigate the effect of hibernation on patterns of DNAm by microarray profiling over 37 000 conserved CpG sites [24]. Many of these sites exhibit reliable change in DNAm with age and, as a consequence, DNAm can be used to accurately predict chronological age in many bat species [25]. In addition, some sites show differences between short and long-lived species in the rate of change of DNAm [25], suggesting that DNAm profiles can provide information regarding the rate of ageing, as has been demonstrated for humans [26–28]. To assess the influence of hibernation on DNAm profiles, we used tissue samples taken in the winter and summer from bats that undergo natural annual hibernation. We used these data to determine if epigenetic age, as predicted by a multi-species bat epigenetic clock, differs between summer and winter samples, and to identify sites in the genome that exhibit change in DNAm associated with hibernation independent of age. Given the results of prior gene expression studies, we hypothesize that animals experience reduced epigenetic ageing during hibernation and that CpG sites that exhibit change in DNAm during hibernation are near genes associated with metabolism and immunity. In addition, we test if CpG sites differentially methylated and associated with hibernation are independent of sites associated with differences in bat longevity [25].

## 2. Methods

The *E. fuscus* sampled in this study were housed in a captive research colony at McMaster University in Hamilton, Ontario, Canada (43.26° N, −79.92° W). Bats in the colony live in an indoor free-flight area (2.5 × 1.5 × 2.3 m) with year-round access to a larger (2.5 × 3.8 × 2.7 m) outdoor flight enclosure [29]. Colony temperature and lighting varied with ambient conditions; however, in the winter, a heater kept the indoor colony temperature above 6°C. Bats have ad libitum access to water and food (yellow mealworms, *Tenebrio molitor*, Reptile Feeders, Norwood, ON). Minimum ages were available for six animals caught as adults 6–9 years before sampling and exact ages for 14 animals born in captivity. When first sampled, these 20 animals ranged from 0.7 to 10.1 years of age. Twelve bats were sampled in February 2020, 20 bats in July 2020 and seven additional bats in February 2021. We excluded one sample from five of the wild-caught animals because of illness at sampling, which left 15 matched pairs from the same animal (12 winter–summer, 3 summer–winter). Big brown bats in Ontario typically initiate hibernation in late October and leave hibernation in early April although animals periodically arouse every 30–45 days throughout the winter to drink and eliminate metabolic waste. Thus, winter samples were taken from captive animals during the hibernation period and summer samples were from the active period.

We used a circular, sterile Sklar Tru-Punch biopsy tool to excise one or two 3-mm diameter tissue biopsies from the wing membrane of each bat. Genomic DNA was extracted from wing tissue using a Zymo Quick-DNA miniprep plus kit (ZymoResearch, Orange, CA, USA) using the standard tissue protocol. Samples with at least 250 ng of DNA but with a concentration below 10 ng µl^−1^ were concentrated using a 30 kDa centrifugal filter to reach at least 12.5 ng µl^−1^ in 20 µl (MilliporeSigma, Burlington, MA, USA).

DNA samples were processed at the UCLA Neuroscience Genomics core facility to measure DNAm. After bisulfite conversion, samples were hybridized to a custom Illumina microarray (HorvathMammalMethylChip40) containing 37 492 probes of conserved 50 base pair sequences with terminal CpG sites. The microarray was designed to assay DNAm from any mammal using probes largely conserved across 62 mammal species [24]. Alignment of the microarray probe sequences to the *E. fuscus* genome (v. 1.0) identified genomic positions for 32 217 CpG sites [25]. These sites were categorized depending on the nearest transcription start site (TSS) for annotated genes as being in either intergenic, 3′ UTR (untranslated region), 5′ UTR, promoter (minus 10 kb to plus 1000 bp from the nearest TSS), exon or intron regions. The proportion of DNA molecules methylated at each CpG site (i.e. Beta values) were obtained after normalization using the SeSaME procedure [30] to correct for bias or variation among plates.

We tested if hibernation influences epigenetic ageing by using a multi-species epigenetic clock created for 712 known-aged bats from 26 species [25]. As noted above, we had minimum ages for 6 of 20 bats (30%). We independently estimated the ages of these animals using a species-specific epigenetic clock created using ‘glmnet’ in R [31], which fitted an elastic net regression model using an alpha of 0.5 between DNAm beta values and chronological age for 60 previously profiled, known-aged *E. fuscus* [25]. The estimated ages were, on average, within 1 year of the minimum ages. We then calculated the residuals between the multi-species clock predicted age and the chronological age of each individual, and compared those residuals between winter (hibernating) and summer (non-hibernating) samples using a linear mixed model, with season and birthplace (captive or wild) as fixed effects and bat identity as a random effect fitted by restricted maximum likelihood. Summary data are reported as mean ± standard error.

To determine if hibernation influences DNAm independent of age at each CpG site, we fitted linear models for DNAm on age using the lm function in R [32] for all *E. fuscus* samples. We then used the resulting residuals in a linear mixed model to compare winter and summer age-adjusted DNAm measured from the same individual with individual included as a random effect. To correct for multiple testing, we used a Benjamini–Yekutieli (BY) false discovery rate (FDR) to obtain adjusted *p*-values [33]. Significant CpG sites (BY *p* < 0.05) are referred to as differentially methylated positions (DMPs). Because this analysis compared age-adjusted DNAm in winter to summer, sites with higher DNAm in the winter are referred to as ‘winter-up’ and sites with lower DNAm in the winter are referred to as ‘winter-down’ (see figure 2 for examples).

We used contingency tests to determine if winter-up or winter-down DMPs were non-randomly distributed in the genome relative to genomic regions and conducted an analysis of variance on the standardized regression coefficients to determine if the magnitude of the effect differed among genomic regions. We compared distance to TSSs between significant and nonsignificant sites using a Student's *t*-test. We also conducted enrichment analyses on DMPs using experimentally derived functional element overlap analysis of ReGions from EWAS, eFORGE v. 2.0 [34]. This programme tests for enrichment of functional regulatory elements by identifying CpG probe sequences associated with DNAse hypersensitive sites, each of five different histone marks or 15 inferred chromatin states identified in human cell lines derived from multiple tissue sources by the Epigenomics Roadmap Consortium (https://egg2.wustl.edu/roadmap/web_portal/). By comparing DMPs to chromatin state or histone associations in human cell lines, eFORGE allows for tissue-specific enrichment tests of DNAm. Permutation tests are then run against the species' genomic background to determine which functional elements occur non-randomly [34]. We conducted separate analyses for winter-up and winter-down DMPs only using sites with an adjusted *p*-value below 0.05 or the most significant 1000 sites in each direction if there were more than 1000 significant sites. As background for the enrichment analysis we selected *E. fuscus* with a 1 kb proximity window. These options constrain the analysis to the 32 217 sites on the 37 K array that have been mapped to the *E. fuscus* genome and use only one site within 1000 bp on each of 1000 permutations. As bat wing tissue is comprised of skin, muscle and blood [35], we only report results for the cell lines derived from similar tissues. For the histone mark analysis there were 7 blood cell lines, 3 fetal muscle cell lines and 4 foreskin cell lines while the chromatin state analysis used 27 blood cell lines, 12 muscle cell lines and 10 skin cell lines. We used a BY FDR of 1% to identify enriched regulatory elements.

We then conducted several association tests to determine the function of genes closest to hibernation DMPs. As multiple probes on the array often are located nearest the same gene, the DNAm effect direction on each gene was determined based on the number of winter-down or winter-up DMPs. Genes with a greater number of significant winter-down sites were considered to have reduced DNAm in winter and genes with a greater number of winter-up sites were considered to have elevated DNAm in winter. Genes with an equal number of winter-up and winter-down DMPs were not considered to have a significant directional effect. We considered all 4808 known genes nearest to mapped CpG sites in the *E. fuscus* genome annotation as background and then used PANTHER v. 16.0 [36] to test for enrichment of winter-up and winter-down genes with respect to protein class, cellular component or biological process using a Fisher's exact test (FET, FDR < 0.05). In a prior study using the same array platform, a total of 1491 CpG sites (corresponding to 700 *E. fuscus* genes) exhibited significant differences in the rate of DNAm change between three long-lived and two short-lived bat species [25]. We used FETs to compare winter-up and winter-down genes to this list of 700 longevity genes and to 4723 innate immunity genes (downloaded from https://www.innatedb.com, 14 August 2020) to determine if hibernation genes are also involved in innate immunity.

## 3. Results

### (a) Hibernation slows epigenetic ageing

**Figure 1.**
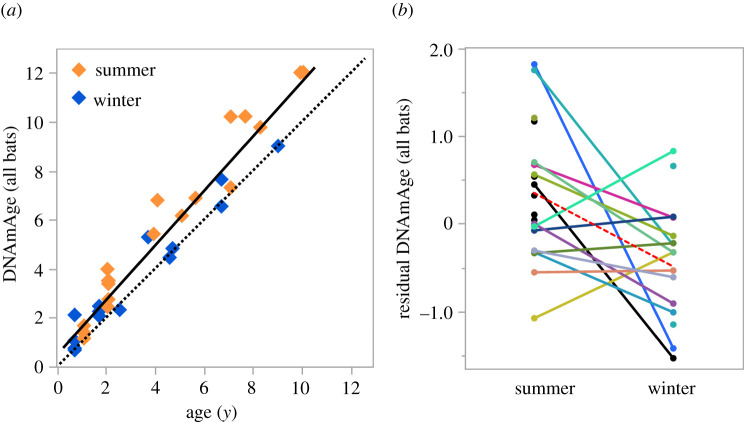
Multi-species epigenetic clock applied to *E. fuscus* sampled in summer and winter. (*a*) Least-squares fit (*solid line*) between chronological age and biological age as predicted by a 26 bat species epigenetic clock from Wilkinson *et al*. [25] with the predicted chronological age indicated by the *dashed y* *=* *x line*. (*b*) Residuals from the regression in (*a*) plotted for each non-hibernating (summer) and hibernating (winter) bat. Solid lines connect individuals with wild-born animals labelled black. The dashed line indicates the change in mean values across seasons. (Online version in colour.)

A multi-species epigenetic clock [25] predicted ages that correlate highly with the chronological ages of big brown bats (*r*^2^ = 0.941, median absolute error (MAE) = 0.531), although the predicted age is equal to or greater than the chronological age in most cases (figure 1*a*). Moreover, the residuals from a regression of predicted epigenetic age on chronological age differ between summer and winter samples (*F* = 7.94, d.f. = 1, 23, *p* = 0.0097), but not birthplace (*F* = 0.13, d.f. = 1, 30, *p* = 0.72), and reveal that the epigenetic age of hibernating bats is 0.77 ± 0.25 years less than the epigenetic age of non-hibernating bats (figure 1*b*).

### (b) Hibernation primarily increases DNAm across the genome

**Figure 2.**
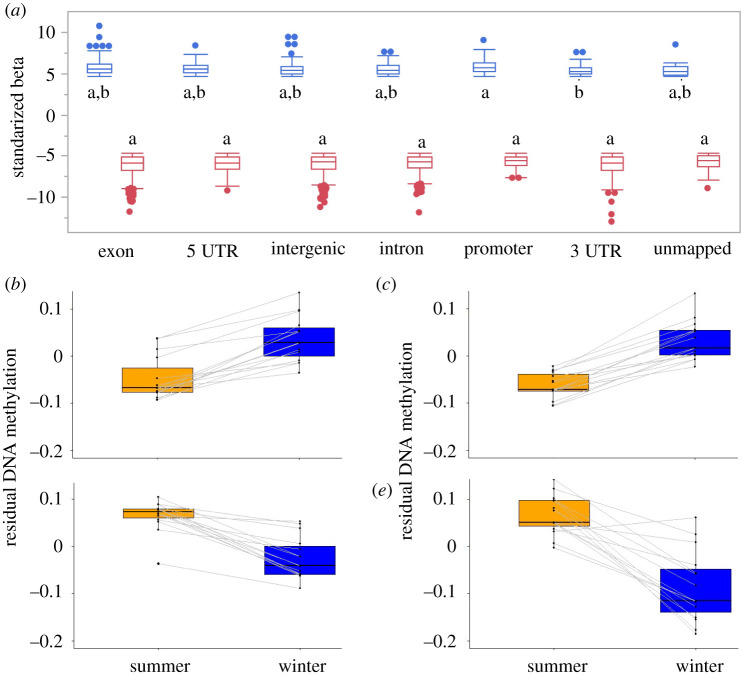
(*a*) Magnitude of the effect of hibernation on significant DMPs among genomic regions, as indicated by box plots showing median, quartiles, whiskers (1.5 times the interquartile range) and outliers of the standardized beta coefficient. Winter-up DMPs in blue, winter-down DMPs in red. Letters indicate results of a Tukey's *post hoc* test with alpha = 0.05. Example winter-up DMPs include (*b*) cg10775708 in an intergenic region near *MAZ* (MYC-associated zinc finger protein) and (*c*) cg05808663 in the promoter region of *NFIX* (Nuclear Factor I X). Example winter-down DMPs include (*d*) cg03445006 in an intergenic region near *BNC2* (Basonuclin 2) and (*e*) cg01905984 in the promoter region of *PHACTR2* (Phosphatase and Actin Regulator 2). In (*b–e*), lines connect samples from the same individual. (Online version in colour.)

A total of 3002 CpG sites mapped (and 78 unmapped) in the *E. fuscus* genome showed significant differences in age-adjusted DNAm across seasons with significantly more winter-up (77.5%) than winter-down DMPs (*X*^2^ = 415.5, *p* = 2.34 × 10^−92^). Hibernation DMPs were widely distributed on 195 of 361 genomic scaffolds but not distributed at random with respect to genomic region (*X*^2^ = 336.7, *p* = 3.28 × 10^−75^). Significant DMPs were further from TSSs than nonsignificant sites in absolute base pair distance (112 021 ± 2879 versus 82 334 ± 7966 bp; *t* = 9.94, *p* < 0.0001), but winter-down DMPs were closer than winter-up DMPs (85 607 ± 5445 versus 119 712 ± 3347 bp; *t* = 5.33, *p* < 0.0001) to TSSs. Corresponding to those genomic locations, more DMPs than expected were located in introns (908 observed/711.8 expected = 1.28; *X*^2^ = 54.1, *p* = 1.92 × 10^−13^) and intergenic regions (956/698.5 = 1.37; *X*^2^ = 95.0, *p* = 1.94 × 10^−22^, but fewer DMPs than expected were located in exons (803/1024.6 = 0.78; *X*^2^ = 47.9, *p* = 4.44 × 10^−12^), promoter regions (119/202.7 = 0.59; *X*^2^ = 34.5, *p* = 4.18 × 10^−9^) and 5′UTRs (108/238.3 = 0.45; *X*^2^ = 71.2, *p* = 3.21 × 10^−17^). DMPs in 3′UTR regions were not enriched (108/126.2 = 0.86; *X*^2^ = 2.64, *p* = 0.10). By contrast, the magnitude of the effect due to the difference in residual DNAm between winter and summer at DMPs showed only minor variation among genomic regions at sites with either decreased (*F* = 2.14, d.f. = 6,687, *p* = 0.047) or increased (*F* = 3.33, d.f. = 6 2379, *p* = 0.003) methylation during winter with the only significant difference revealed by *post hoc* tests occurring between winter-up sites at promoters and 3′UTR regions (figure 2*a*).

### (c) DNAm changes associated with hibernation influence metabolic processes, immunity and longevity

**Figure 3.**
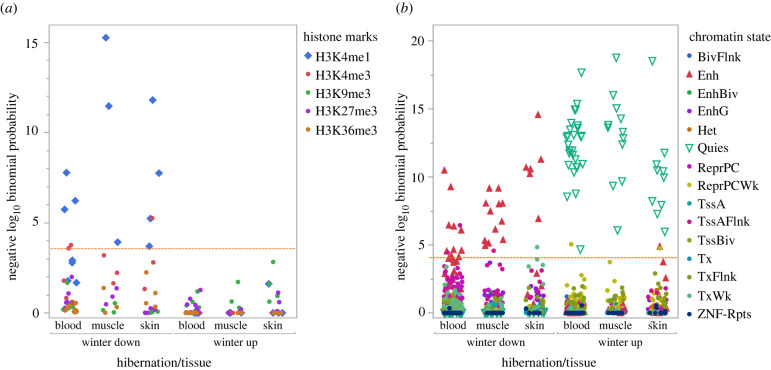
Evidence of tissue-specific epigenomic enrichment obtained from eFORGE for (*a*) 5 histone marks across 7 blood cell lines, 3 muscle cell lines and 4 skin cell lines and (*b*) 15 chromatin states across 14 blood cell lines, 6 muscle cell lines and 8 skin cell lines. Chromatin state abbreviations are BivFlnk, flanking bivalent TSS/Enh; Enh, enhancers; EnhBiv, bivalent enhancer; EnhG, genic enhancers; Het, heterochromatin; Quies, quiescent/low; ReprPC, repressed PolyComb; ReprPCWk, weak repressed PolyComb; TssA, active TSS; TssAFlnk, flanking active TSS; TssBiv, bivalent/poised TSS; Tx, strong transcription; TxFlnk, transcription at gene 5′ and 3′; TxWk, weak transcription; ZNF/Rpts, zinc finger genes and repeats. *Red dashed line* indicates significance (BY adjusted *p* < 0.01). (Online version in colour.)

An eFORGE analysis revealed that one histone mark, H3K4me1, which is often associated with active or primed enhancers, was enriched in multiple cell lines from all three tissue sources in winter-down DMPs, but no histone mark was significantly associated with winter-up DMPs (figure 3*a*). The eFORGE analysis of inferred chromatin states using winter-down DMPs also indicates significant enrichment for enhancers in cell lines derived from blood, skin and muscle tissues while winter-up DMPs were highly enriched for quiescent states in cell lines from all three tissue sources (figure 3*b*).

**Figure 4.**
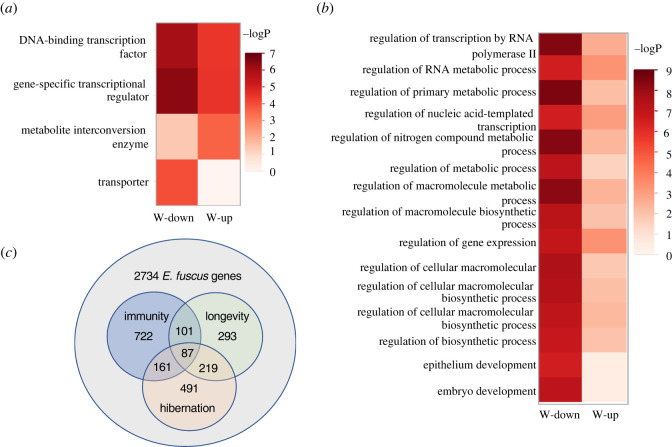
Gene ontology enrichment analysis for (*a*) protein class and (*b*) biological process. Significance from a Fisher's exact rest is indicated by colour in each cell. The threshold 1% FDR is –log_10_ *p* = 3.5. (*c*) Number and overlap of genes nearest significant DMPs associated with hibernation, innate immunity and longevity. (Online version in colour.)

Winter-up DMPs were nearest 739 genes and winter-down DMPs were nearest 219 genes. Gene ontology enrichment tests for cellular components revealed that genes associated with chromatin were significantly enriched for winter-up and winter-down genes. Winter-down genes were also significantly enriched for nuclear, chromosomal and non-membrane-bounded organelle cell components. Winter-down and winter-up genes were enriched for gene specific transcription regulators and transcription factors (figure 4*a*). Indeed, over 40 biological processes were significant after adjusting for multiple testing. The top 15 significant biological processes were associated with winter-down genes and 13 of those were associated with regulation of metabolism or transcription (figure 4*b*).

Significant overlap was also found between innate immunity genes and genes nearest hibernation DMPs (figure 4*c*). Of the 1071 innate immunity genes with sites on the array, 248 (23.2%) exhibited evidence of differential methylation (*X*^2^ = 9.02, *p* = 0.0027), a majority of which (188; 75.8%) were associated with elevated DNAm during hibernation. Comparison of 700 previously identified bat longevity genes to the 958 hibernation genes reveals 306 genes in common (figure 4*c*)—dramatically more than expected by chance (*X*^2^ = 290.6, *p* = 3.68 × 10^−65^), with 52 (17%) longevity genes associated with winter-down DMPs. Moreover, 41 genes nearest winter-down DMPs and associated with bat longevity exhibit differential gene expression during hibernation or torpor in other mammals and six have been previously identified as candidate torpor genes (electronic supplementary material, table S1).

## 4. Discussion

Application of a multi-species bat epigenetic clock [25] provides strong evidence that hibernation is associated with slower epigenetic ageing. The multi-species clock explains 94% of the variation in the chronological ages of both hibernating and non-hibernating big brown bats; however, the clock estimates are equal to or greater than the chronological age, suggesting big brown bats age slightly faster than a ‘typical’ bat, especially during the active period (figure 1). The multi-species bat clock was created using DNAm data from 26 species, some of which have longer lifespans for their body sizes than big brown bats (cf. figure 2, [25]). Moreover, comparison of deviations from a regression of predicted on true ages reveals that the epigenetic age of hibernating bats is nearly a year less than non-hibernating bats after adjusting for age. This finding provides direct evidence that hibernation is associated with reduced epigenetic ageing in bats (Order Chiroptera), and is consistent with a recent study in marmots (Order Rodentia) that reached the same conclusion [21]. Because the multi-species clock only uses DNAm from 162 CpG sites, this result does not reveal the extent to which hibernation influences DNAm in the genome. After adjusting for age, comparison of DNAm at over 32 000 sites profiled in samples taken from the same animal during the winter hibernation period versus the summer active season reveals that hibernation alters DNAm at over 3000 DMPs widely distributed in the genome of *E. fuscus.* Over three times as many DMPs exhibited increased DNAm as opposed to decreased DNAm during hibernation. Given that elevated DNAm is typically associated with suppressed gene transcription, this result is consistent with a reduction in the activity of many genes during hibernation.

The amount of DNAm can vary by tissue; however, the eFORGE analysis revealed evidence of consistent enrichment of histone marks and inferred chromatin states in multiple human cell lines derived from blood, skin and muscle tissues (figure 3). Winter-up DMPs were not associated with any histone marks but were highly associated with quiescent chromatin states in all cell lines. Given that quiescence is a stress-resistant state in which cells suspend most processes, this finding is consistent with reduced metabolism and immune function during hibernation [6,37]. By contrast, winter-down DMPs were enriched for H3K4me1, a histone mark associated with enhancers [38] and enriched for enhancer chromatin states. In other words, winter-down DMPs were associated with active gene transcription. We suspect these results provide a conservative estimate of the number of regulatory factors involved both because the array only samples a fraction of possible enhancer regions and because enhancer motifs can differ among mammals [39]. Nevertheless, the strength and consistency of the evidence for enhancer enrichment across multiple cell lines indicates there must be substantial sequence conservation at these regions.

Enrichment analysis of winter-up genes failed to detect significant enrichment for any biological process, indicating that elevated DNAm during hibernation is associated with a diverse ensemble of genes in proportion to their relative abundance on the array. By contrast, enrichment analysis of winter-down genes found evidence for enrichment of DNA-binding transcription factors, especially those involved in regulating metabolic processes (figure 4). This result is consistent with the eFORGE analysis and with previous findings on squirrels and marsupials that found increased expression of genes involved in cold-tolerant mechanisms of metabolism and regulation of pathways involved in protein turnover [5,40] or cryoprotection [5,41] during hibernation.

In addition to being associated with regulating metabolic processes, genes nearest to hibernation DMPs were also more likely to be involved in innate immunity and bat longevity as indicated by different age-dependent rates of change in DNAm between short-lived and long-lived bats [25]. Most (79%) of the 52 genes nearest winter-down DMPs associated with bat longevity exhibit differential gene expression during torpor in other mammals (electronic supplementary material, table S1). These results are consistent with longevity in bats being associated with periodic episodes of reduced energy consumption due to lowered metabolism and suppressed immune function. This study illustrates how DNAm profiling using nonlethal tissue samples can provide real-time insight into the epigenetic control of physiological processes, such as hibernation.

## Data Availability

All data used in this study are freely available. Normalized methylation values for each sample, along with sample metadata, are available from NCBI as series GSE164127 (https://www.ncbi.nlm.nih.gov/geo/query/acc.cgi?acc=GSE164127). The design of the Illumina microarray (HorvathMammalMethylChip40) is available from NCBI as platform GPL28271 (https://www.ncbi.nlm.nih.gov/geo/query/acc.cgi?acc=GPL28271). R scripts, clock coefficients, age predictions, probe annotations for the *E. fuscus* genome, eFORGE output and statistics for identifying DMPs are available at doi:10.6084/m9.figshare.c.5927288.v1 [42]. Electronic supplementary material is available online [43].
